# Neutropenia and G-CSF in lymphoproliferative diseases

**DOI:** 10.1179/1607845412Y.0000000049

**Published:** 2013-05

**Authors:** Roberto Ria, Antonia Reale, Michele Moschetta, Franco Dammacco, Angelo Vacca

**Affiliations:** Department of Biomedical Sciences and Human Oncology, University of Bari ‘Aldo Moro’ Medical School, Bari, Italy

**Keywords:** Chemotherapy, G-CSF, Neutropenia, Non-Hodgkin's lymphoma, Hodgkin's lymphoma, Multiple myeloma

## Abstract

**Background:**

Chemotherapy-induced neutropenia is a major cause of morbidity and mortality. It frequently causes dose reductions or treatment delay, which can be prevented or treated by the administration of granulocyte-colony-stimulating factor (G-CSF). However, a better knowledge of the incidence, day of onset after therapy, and duration of neutropenia is essential to optimize the use of G-CSF.

**Design and methods:**

Six hundred and ninety-four patients from a single institution, affected by lympho-proliferative diseases, were retrospectively reviewed for the occurrence of grade 4 neutropenia and febrile neutropenia (FN). Duration of neutropenia and time of neutrophil nadir were also retrieved. The diagnoses included non-Hodgkin's lymphoma, Hodgkin's lymphoma, and multiple myeloma. Chemotherapy regimens were obviously different according to the diagnosis, disease stage, and first or subsequent lines of therapy.

**Results:**

No patient received G-CSF as primary prophylaxis. Median nadir did not significantly differ among patients treated with first or successive lines of therapy. The incidence of grade 4 neutropenia and FN ranged from 0 to 94%, depending on the chemotherapy regimen. Patients receiving a first-line chemotherapy regimen had a significantly lower incidence of febrile grade 4 neutropenia compared to patients treated with a second or subsequent line of therapy. The duration of grade 4 neutropenia was significantly longer in patients given second or subsequent lines.

**Conclusion:**

The results of this study could be useful to define the nadir onset in the hematologic setting in order to correctly tailor timing and duration of G-CSF prophylaxis and to assess the lowest fully effective dose.

## Introduction

Chemotherapy-induced neutropenia is one of the most relevant causes of morbidity and mortality.^1,2^ Despite the wide availability of effective antibiotics, febrile neutropenia (FN) remains a life-threatening medical emergency and is associated with remarkable social costs^1,2^. Dose reduction and treatment delay are common consequences that may limit the efficacy of the treatment,^3,4^ given that completion of all planned chemotherapy cycles is essential to provide patients with the maximum chance of treatment success.^3,4^

The chemotherapy regimen is one of the primary determinants of the risk of neutropenia, given that some regimens are more myelotoxic than others. For example, the combination of cyclophosphamide, methotrexate, and 5-fluorouracil is less toxic than adriamycin plus cyclophosphamide or combined cyclophosphamide, doxorubicin, and 5-fluorouracil. High cyclophosphamide doses or the use of etoposide and high anthracycline doses have also been identified as significant predictors of severe neutropenia and FN.^5^ Additional risk factors of neutropenia are the intensity of specific chemotherapy regimens and the phase of therapy. Indeed, the highest risk has been noticed in the initial cycles with the demonstration that 65% of the hospitalizations for FN occurred in the first two cycles of chemotherapy.^6,7^

It has been clearly established that in patients receiving myelotoxic chemotherapy regimens, prophylaxis with granulocyte-colony-stimulating factor (G-CSF) decreases the occurrence of FN.^8^ Indeed, recombinant human G-CSF enhances proliferation and differentiation of neutrophils and helps to ensure a correct dose intensity and dose density.

According to international guidelines, in settings characterized by a high risk of FN (20% or more), prophylaxis with G-CSF 5 µg/kg/day subcutaneously (s.c.) should last 24–72 hours after chemotherapy until a post-nadir recovery of absolute neutrophil count (ANC) has been achieved.^9–11^ Alternatively, a single 6 mg s.c. injection of peg-filgrastim, administered 24 hours after chemotherapy, is comparable to 11 daily injections of G-CSF in terms of neutrophil recovery.^12–14^ The onset and duration of nadir are key points to establish starting day and duration of G-CSF administration after chemotherapy.^15^

Attempts to establish alternative G-CSF scheduling in moderate- and high-intensity chemotherapy regimens^16–23^ suggest that reduction in the number of G-CSF injections is feasible without substantially changing the outcome. A shorter schedule seems to decrease the incidence and severity of side effects and is more cost-effective.^21,22^ In addition, a large survey by Falandry *et al.*^24^ on the use of G-CSF in clinical practice has recently emphasized that compliance with international guidelines results in its suboptimal prescription. In particular, this observational study indicates that the required duration of daily G-CSF administration is significantly shorter than recommended in guidelines, namely 5.5 days, whereas only 9.3% of the patients exceed 7 days. It has also been assessed that about 96% of G-CSF injections concern clinical situations for which G-CSF is not recommended by current guidelines.^25,26^ Possible explanations for the discrepancies between guidelines and clinical practice could be that guidelines are often based on studies designed to ascertain the efficacy of G-CSF versus placebo, regardless of proper timing and duration.

The aim of this paper is to define the time of onset and the duration of neutrophil nadir following chemotherapy and to clarify the possible differences between first and second or subsequent lines of treatment.

## Patients and methods

### Patients

Between January 1995 and December 2011, 694 patients were retrospectively reviewed. Patients had undergone chemotherapy for non-Hodgkin's lymphoma (NHL), Hodgkin's lymphoma (HL), and multiple myeloma (MM). Patients who were assigned to receive high-dose chemotherapy were excluded from the study.

### Design

Patients were stratified according to the hematological malignancy. Chemotherapy regimens used in at least 10% of the patients were considered for each group. The following endpoints were considered: (a) nadir onset, defined as the day of the lowest neutrophil count; (b) incidence of grade 4 neutropenia, defined as the proportion of patients experiencing ANC < 500/mm^3^; (c) duration of grade 4 neutropenia, defined as the number of days from onset to resolution of grade 4 neutropenia; (d) incidence of FN, defined as the proportion of patients experiencing ANC < 1000/mm^3^ and a single temperature value >38.3°C (101°F) or a sustained daily temperature of ≥38°C (100.4°F) for more than 1 hour; (e) duration of FN, defined as the number of days from its onset to resolution.

All these data were evaluated according to treatment line (first or second/subsequent line). The use of G-CSF as primary or secondary prophylaxis and the possible use of antibiotic therapy were also considered. Finally, reduction of chemotherapy dose, delay in the chemotherapy administration, or the need for hospitalization was evaluated for each cycle.

The study, which was approved by the local Ethical Committee, was conducted according to the Declaration of Helsinki principles.

### Statistical analysis

Descriptive statistics refer to all included patients. For continuous variables, mean, minimum, and maximum values were calculated. For each discrete variable, the number of cases in each category was calculated.

Data were analyzed with the SPSS (Chicago, IL, USA) software package. All results are presented as median ± 1 SD (range). Medians were compared with the Mann–Whitney *U*-test. *P* values <0.05 were considered significant. One-way analysis of variance analysis was used to compare all parameters in patients with NHL, HL, and MM, taking into account neutropenia values and line(s) of therapy.

## Results

Out of 694 patients included in this analysis, 176 had NHL, 127 HL, and 391 MM. Patients characteristics are listed in Tables 1–3.

**Table 1:** Characteristics of NHL patients

	Population (*n* = 176) % (*N*)	
Age (years)		
Median	57	
Range	(29–91)	
Gender		
Male/female	91/85	
Histology		
B-/T-cell	74 (131)/26 (45)	
High grade/low grade	58 (103)/42 (73)	
IPI		
1	27 (48)	
2	38 (66)	
3	35 (62)	
Bulky	22 (39)	
Chemotherapy regimens	1st line	≥ 2nd line
R-CHOP	48 (83)	13 (35)
CHOP	14 (25)	6 (16)
FND	8 (14)	9 (24)
R-CVP	5 (10)	4 (12)
IEV	3 (6)	25 (70)
DHAP	15 (26)	24 (68)
PROMACE-CyTABOM	7 (12)	11 (31)
ESHAP	0	8 (21)
Radiotherapy	42 (74)	

NHL: non-Hodgkin's lymphoma; *N*: number of patients; IPI: International Prognostic Index; R-CHOP: rituximab, cyclophosphamide, doxorubicin, vincristine, prednisone; CHOP: cyclophosphamide, doxorubicin, vincristine, prednisone; FND: fludarabine, mitoxantrone, dexamethasone; R-CVP: cyclophosphamide, vincristine, prednisone, rituximab; IEV: ifosfamide, etoposide, epirubicin; DHAP: dexamethasone, cisplatinum, cytarabine; PROMACE-CyTABOM: cyclophosphamide, doxorubicin, etoposide, prednisone, cytarabine, bleomycin, vincristine, methotrexate, folic acid; ESHAP: etoposide, methylprednisolone, cytarabine, cisplatinum.

**Table 2:** Characteristics of HL patients

	Population (*n* = 127) % (*N*)	
Age (years)		
Median	34	
Range	(18–72)	
Gender		
Male/female	48/79	
Histology		
SN	41 (52)	
MC	16 (21)	
LP	31 (39)	
LD	12 (15)	
IPI		
0–1	18 (23)	
2–3	44 (56)	
4–5	38 (48)	
Bulky	45 (57)	
Chemotherapy regimens	1st line	≥ 2nd line
C-MOPP/ABVD	25 (32)	0
ABVD	56 (71)	8 (12)
MOPP	19 (24)	23 (32)
BEACOPP	0	30 (42)
Enhanced BEACOPP	0	13 (18)
IGEV	0	26 (36)
Radiotherapy	66 (84)	

HL: Hodgkin's lymphoma; *N*: number of patients; IPI: International Prognostic Index; ABVD: doxorubicin, bleomycin, vinblastine, dacarbazine; MOPP: mecloretamine, vincristine, procarbazine, prednisone; BEACOPP: bleomycin, etoposide, doxorubicin, cyclophosphamide, vincristine, procarbazine, prednisone; enhanced BEACOPP: bleomycin, etoposide, doxorubicin, cyclophosphamide, vincristine, procarbazine, prednisone; IGEV: ifosfamide, gemcitabine, vinorelbine, prednisone.

**Table 3:** Characteristics of multiple myeloma patients

	Population (*n* = 391) % (*N*)	
Age (years)		
Median	64	
Range	(37–86)	
Gender		
Male/female	209/182	
Renal failure		
Yes	32 (124)	
No	68 (267)	
D&S stage		
1	5 (18)	
2	24 (94)	
3	71 (279)	
Chemotherapy regimens	1st line	≥ 2nd line
MP	23 (88)	23 (194)
MPT	16 (64)	13(102)
V-MP	24 (76)	3 (21)
TD	12 (47)	11 (95)
VD	7 (28)	8 (66)
Rd	4 (14)	10 (87)
V-MPT	4 (14)	0
VAD	10 (41)	4 (36)
VBAP	1 (2)	5 (44)
VMCP	1 (3)	10 (86)
Vinorelbine/dexamethasone	0	3 (21)
Intermediate-dose Cyclophosphamide	2 (6)	10 (86)

MM: multiple myeloma; *N*: number of patients; D&S stage: Durie and Salmon stage; MP: melphalan, prednisone; MPT: melphalan, prednisone, thalidomide; V-MP: bortezomib, melphalan, prednisone; TD: thalidomide, dexamethasone; VD: bortezomib, dexamethasone; Rd: lenalidomide, dexamethasone; V-MPT: bortezomib, melphalan, prednisone, thalidomide; VAD: vincristine, doxorubicin, dexamethasone; VBAP: vincristine, carmustine, doxorubicin, prednisone; VMCP: vincristine, melphalan, cyclophosphamide, prednisone.

The most commonly used chemotherapy regimens were R-CHOP (rituximab, cyclophosphamide, doxorubicin, vincristine, prednisone) as first-line treatment, IEV (ifosfamide, etoposide, epirubicin) and DHAP (dexamethasone, cisplatinum, cytarabine) as second/subsequent lines for NHL patients; ABVD (doxorubicin, bleomycin, vinblastine, dacarbazine) as first line, BEACOPP (bleomycin, etoposide, doxorubicin, cyclophosphamide, vincristine, procarbazine, prednisone) and IGEV (ifosfamide, gemcitabine, vinorelbine, prednisone) as second/subsequent lines for HL patients; MP (melphalan, prednisone) and V-MP (bortezomib, melphalan, prednisone) as first line, MPT (melphalan, prednisone, thalidomide), Len/Dex (Lenalidomide, Dexamethasone), and MP (melphalan, prednisone) as second/subsequent line for MM patients. None of the patients received primary prophylaxis with G-CSF, according to our routine procedure in our department.

**Figure 1:**
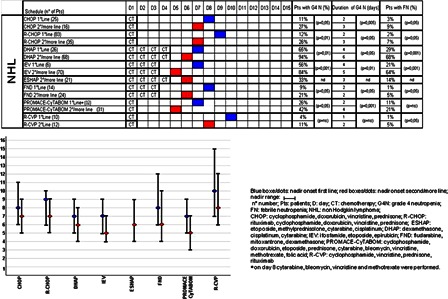
For every chemotherapy regimen length of chemotherapy administration (CT), nadir onset (blue boxes/dots for first line, red boxes/dots for second/more line), and ranges, incidence of grade 4 neutropenia, duration of grade 4 neutropenia, incidence of FN are reported. Variables are considered and compared in first and second/more line.

**Figure 2:**
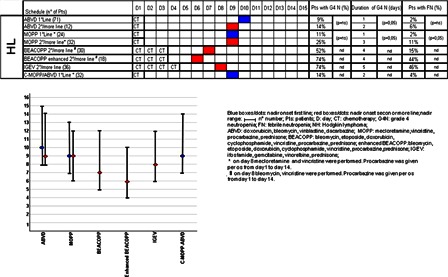
For every chemotherapy regimen length of chemotherapy administration (CT), nadir onset (blue boxes/dots for first line, red boxes/dots for second/more line), and ranges, incidence of grade 4 neutropenia, duration of grade 4 neutropenia, incidence of FN are reported. Variables are considered and compared in first and second/more line.

**Figure 3:**
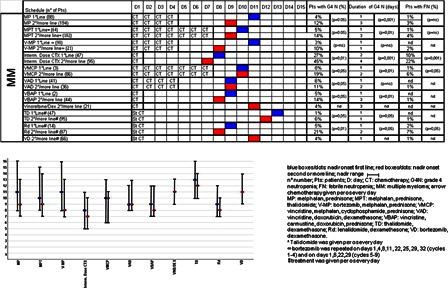
For every chemotherapy regimen length of chemotherapy administration (CT), nadir onset (blue boxes/dots for first line, red boxes/dots for second/more line), nadir range, incidence of grade 4 neutropenia, duration of grade 4 neutropenia, incidence of FN are reported. Variables are considered and compared in first and second/more line.

The onset of nadir, reported in Figs. 1–3, shows no difference between first and second/subsequent lines for all the regimens considered. The incidence of grade 4 FN varied from 0 to 94% (Figs. 1–3). As expected, the incidence of grade 4 neutropenia and FN was greater in patients treated with IEV and DHAP in NHL, in patients treated with BEACOPP, enhanced BEACOPP and IGEV in HL, whereas these complications were less frequent in MM.

The incidence of neutropenia and FN following second or subsequent lines was significantly higher for all chemotherapeutic regimens compared with patients treated in first line (Figs. 1–3). In particular, all regimens used in NHL as first-line treatment were associated with neutropenia in percentages <20% except for IEV, DHAP, and PROMACE CyTABOM; all regimens used in first-line patients affected by HL had <20% FN and all patients affected by MM had <20% neutropenia, except for intermediate dose cyclophosphamide (ICTX).

Median duration of neutropenia ranged from 1 to 6 days and was longer in patients treated with chemotherapy regimens characterized by a greater incidence of neutropenia (PROMACE CyTABOM, BEACOPP, enhanced BEACOPP, DHAP, IEV). Patients under first-line treatment had a significantly shorter duration of grade 4 neutropenia compared to patients treated in second or subsequent lines with the same chemotherapy regimen. In particular, all first-line patients, except those affected by NHL treated with DHAP and IEV, had a median duration of neutropenia of less than 2 days.

All patients who experienced grade 4 neutropenia or FN were treated with G-CSF (5 µg/kg/die) until neutropenia improved to grade 2. Moreover, broad-spectrum antibiotics were administered to febrile patients. Secondary prophylaxis with G-CSF was regularly performed at the subsequent cycles. In none of the patients was dose reduction or treatment delay necessary.

## Discussion

Neutropenia is fairly common in patients undergoing chemotherapy. It makes patient's management complex and is associated with increased mortality.^1,2^ The presence of grade 4 neutropenia after chemotherapy and the absence of fever or other signs of infection should alert the clinician to the patient's risk for developing fever and infection. It is recognized widely that profound neutropenia places a patient at very high risk for serious infectious complications. Moreover, significant costs are incurred when FN develops in a patient treated with chemotherapy. These costs include both direct medical costs and indirect costs that are borne by the patient and his or her family.

The administration of G-CSF decreases the incidence of FN^8^ and allows the maintenance of a correct dose density and dose intensity. Although current international guidelines have singled out the chemotherapy regimens which require G-CSF support and the optimal timing of administration,^9–11^ a recent survey has demonstrated that in clinical practice the number of G-CSF vials actually required is about half of those indicated by the guidelines for most patients.^23^ Furthermore, in the oncologic and hematologic settings it has been shown that shorter courses of daily G-CSF are able to prevent neutropenia, reduce incidence of short-term and long-term side effects and result in cost saving.^21–26^

**Figure 4:**
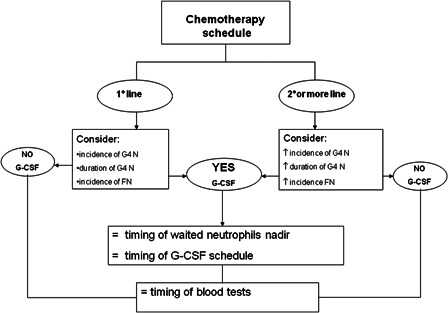
Proposed G-CSF schedule.

Most chemotherapy regimens employed in the chronic hematologic setting were included in this retrospective analysis in order to identify, in the absence of G-CSF administration, the incidence and the duration of grade 4 neutropenia, FN, and the median onset of nadir. These variables were compared in patients receiving first, second, or subsequent lines of therapy. The results indicate that for each chemotherapy regimen the first concern regards the line of treatment and that for each condition the risk of neutropenia, FN, and their duration should be evaluated. If the risk is high, G-CSF should be administered considering the neutrophils nadir. It is important to underscore that nadir onset does not vary among different lines of treatment and this could lead to define a unique G-CSF schedule of treatment for each chemotherapy regimen (Fig. 4). For each chemotherapy schedule considered, the risk of G4 neutropenia or FN remains unchanged (i.e. the onset of nadir is the same in first and second or more lines); patient's risk, on the contrary, varies and increases with the line of treatment (incidence and duration of G4 neutropenia and FN are significantly increased in second or more lines).

In our case series, patients treated in first line with R-CHOP regimen, for example, had a 12% incidence rate of grade 4 neutropenia, a 2% incidence rate of FN, a median nadir duration of 2 days and median nadir onset on day 9 (range from day 6 to day 10). In patients given second or more line treatment, instead, the incidence of both grade 4 neutropenia and FN is significantly increased (26% and 7% respectively) and the median duration is longer (3 days), but nadir onset, even if slightly anticipated, is not statistically different (day 9 and day 7, respectively). These parameters could be useful to tailor correct timing and duration of G-CSF prophylaxis.

## Authorship and disclosures

RR was the principal investigator and takes primary responsibility for the paper. RR, AR, and MM collected and analyzed the data of patients. RR performed the statistical analysis, RR, FD, and AV co-ordinated the research. RR and AR wrote the paper.

## Funding

This work was supported by Associazione Italiana per la Ricerca sul Cancro (AIRC), Investigator Grant and Special Program Molecular Clinical Oncology 5 per thousand (number 9965), Milan, the EU Multiple Myeloma Program FP7 OVER-MyR HEALTH.2011.2.4.1-2 and the Ministry of Health (Progetto PRIN 2009), Rome, Italy.
